# Metabolic Syndrome Derived from Principal Component Analysis and Incident Cardiovascular Events: The Multi Ethnic Study of Atherosclerosis (MESA) and Health, Aging, and Body Composition (Health ABC)

**DOI:** 10.1155/2012/919425

**Published:** 2012-03-21

**Authors:** Subhashish Agarwal, David R. Jacobs, Dhananjay Vaidya, Christopher T. Sibley, Neal W. Jorgensen, Jerome I. Rotter, Yii-Der Ida Chen, Yongmei Liu, Jeanette S. Andrews, Stephen Kritchevsky, Bret Goodpaster, Alka Kanaya, Anne B. Newman, Eleanor M. Simonsick, David M. Herrington

**Affiliations:** ^1^Division of Cardiology, Department of Internal Medicine, Oakwood Hospital and Medical Center, Dearborn, MI 48124, USA; ^2^Department of Cardiology, Wake Forest School of Medicine, Winston-Salem, NC 27157, USA; ^3^Division of Epidemiology and Community Health, School of Public Health, University of Minnesota, Minneapolis, MN 55455, USA; ^4^Division of General Internal Medicine, Department of Medicine, John Hopkins University, Baltimore, MD 21205, USA; ^5^National Institutes of Health, Bethesda, MD 20892, USA; ^6^Department of Epidemiology, University of Washington, Seattle, WA 98195, USA; ^7^Medical Genetics Institute, Cedars-Sinai Medical Center, Los Angeles, CA 90048, USA; ^8^Department of Epidemiology and Prevention, Center for Human Genomics, Wake Forest School of Medicine, Winston-Salem, NC 27157, USA; ^9^Department of Biostatistical Sciences, Wake Forest School of Medicine, Winston-Salem, NC 27157, USA; ^10^Geriatrics & Gerontology, Sticht Center on Aging, Wake Forest School of Medicine, Winston-Salem, NC 27157, USA; ^11^Division of Endocrinology and Metabolism, University of Pittsburgh, Pittsburgh, PA 15260, USA; ^12^Department of General Internal Medicine, University of California, San Francisco, CA 94143, USA; ^13^Departments of Epidemiology and Medicine, University of Pittsburgh, Pittsburgh, PA 15260, USA; ^14^National Institute on Aging Intramural Research Program, Division of Geriatric Medicine and Gerontology, John Hopkins University, Baltimore, MD 21205, USA

## Abstract

*Background*. The NCEP metabolic syndrome (MetS) is a combination of dichotomized interrelated risk factors from predominantly Caucasian populations. We propose a continuous MetS score based on principal component analysis (PCA) of the same risk factors in a multiethnic cohort and compare prediction of incident CVD events with NCEP MetS definition. Additionally, we replicated these analyses in the Health, Aging, and Body composition (Health ABC) study cohort. *Methods and Results*. We performed PCA of the MetS elements (waist circumference, HDL, TG, fasting blood glucose, SBP, and DBP) in 2610 Caucasian Americans, 801 Chinese Americans, 1875 African Americans, and 1494 Hispanic Americans in the multiethnic study of atherosclerosis (MESA) cohort. We selected the first principal component as a continuous MetS score (MetS-PC). Cox proportional hazards models were used to examine the association between MetS-PC and 5.5 years of CVD events (*n* = 377) adjusting for age, gender, race, smoking and LDL-C, overall and by ethnicity. To facilitate comparison of MetS-PC with the binary NCEP definition, a MetS-PC cut point was chosen to yield the same 37% prevalence of MetS as the NCEP definition (37%) in the MESA cohort. Hazard ratio (HR) for CVD events were estimated using the NCEP and Mets-PC-derived binary definitions. In Cox proportional models, the HR (95% CI) for CVD events for 1-SD (standard deviation) of MetS-PC was 1.71 (1.54–1.90) (*P* < 0.0001) overall after adjusting for potential confounders, and for each ethnicity, HRs were: Caucasian, 1.64 (1.39–1.94), Chinese, 1.39 (1.06–1.83), African, 1.67 (1.37–2.02), and Hispanic, 2.10 (1.66-2.65). Finally, when binary definitions were compared, HR for CVD events was 2.34 (1.91–2.87) for MetS-PC versus 1.79 (1.46–2.20) for NCEP MetS. In the Health ABC cohort, in a fully adjusted model, MetS-PC per 1-SD (Health ABC) remained associated with CVD events (HR = 1.21, 95%CI 1.12–1.32) overall, and for each ethnicity, Caucasian (HR = 1.24, 95%CI 1.12–1.39) and African Americans (HR = 1.16, 95%CI 1.01–1.32). Finally, when using a binary definition of MetS-PC (cut point 0.505) designed to match the NCEP definition in terms of prevalence in the Health ABC cohort (35%), the fully adjusted HR for CVD events was 1.39, 95%CI 1.17–1.64 compared with 1.46, 95%CI 1.23–1.72 using the NCEP definition. *Conclusion*. MetS-PC is a continuous measure of metabolic syndrome and was a better predictor of CVD events overall and in individual ethnicities. Additionally, a binary MetS-PC definition was better than the NCEP MetS definition in predicting incident CVD events in the MESA cohort, but this superiority was not evident in the Health ABC cohort.

## 1. Introduction

Metabolic syndrome (MetS) is a constellation of interrelated cardiovascular risk factors that increase the risk of developing both atherosclerotic cardiovascular disease and Type 2 DM [1]. It was recognized as a syndrome in 1988 [2] as it reflects joint action of specific cardiovascular risk factors whose underlying pathophysiology is thought to be related to insulin resistance. Since then, various expert committees have tried to refine the definition of MetS [1, 3–6], and some have concluded that MetS in its current form is imprecisely defined, leads to loss of critical information due to dichotomization of continuous variables [7, 8], and has uncertain value as a cardiovascular disease marker calling for further research in this field. Thus, one school of thought regarding the true nature of MetS asserts that MetS is a representation of the true underlying pathophysiologic pathways of dysmetabolic components [2]. Another viewpoint is that MetS is a purely operational, arbitrary, and convenient scoring methodology that summarizes risk [7, 8]. It is deficient in that it omits smoking and LDL-C and is therefore not as efficient as the Framingham risk score and it does not add new elements [7, 8]. The intention of this paper is to highlight a methodology to improve upon the current NCEP definition of MetS, in the sense of improving CVD risk prediction using the same components as are included in the MetS as currently defined [1].

The commonly used definition of MetS from National Cholesterol Education Program/Adult Treatment Panel (NCEP ATP III) [1, 9] is a score based on the presence or absence of five dichotomized risk factors. When a subject exceeds the cut point for three or more of these factors, the syndrome is deemed to be present [1, 3]. However, it is accepted that the risk for CVD from each element of the MetS is continuous, and dichotomization above and below certain thresholds does not lead to presence or absence of CVD risk. Studies show that dichotomization of the elements of MetS based on ad hoc cut points may lead to misclassification of risk in individuals, despite minimal changes in the actual elements or cardiovascular risk [10, 11]. Further, the summation of components into a unitary diagnosis assumes that each dichotomized risk factor carries the same risk, yet some factors included in NCEP MetS definition are more strongly predictive of CVD than others [8]. Additionally, the NCEP definition of MetS does not account for racial differences and may not accurately predict cardiovascular risk in non-Caucasian populations [12–16].

Principal component analysis is an analysis strategy designed to summarize multidimensional correlated data [17, 18] and provide a continuous MetS score unlike factor analysis [17, 18] which aims to determine the underlying structure of the syndrome by identifying latent variables. The unrotated first principal component is a linear combination of the individual variables that captures the maximum variance in the data among all possible linear combinations. Since MetS is characterized by concomitant derangements in multiple factors, the first principal component can be used to indicate the extent to which any individual's metabolic risk factors are consistent with having the MetS. A few studies have applied PCA to derive a continuous MetS score and how this score relates to incident diabetes and cardiovascular disease (CVD) [19–23]. This study is an extension and validation of such findings in a multiethnic cohort.

## 2. Materials and Methods

### 2.1. Study Populations and Data Collection

 The Multi-Ethnic Study of Atherosclerosis (MESA) design has been previously described [24]. Briefly, MESA is a prospective cohort study that began in July 2000 to investigate the prevalence, correlates, and progression of subclinical CVD. It included 6814 men and women aged 44–84 years old recruited from 6 US communities (Baltimore, MD; Chicago, IL; Forsyth County, NC; Los Angeles County, CA; northern Manhattan, NY; St. Paul, MN). MESA cohort participants were 38% Caucasian (*n* = 2622), 28% African American (*n* = 1893), 22% Hispanic (*n* = 1496), and 12% Chinese (*n* = 803). Individuals with a history of physician-diagnosed myocardial infarction, angina, heart failure, stroke, or transient ischemic attack, or who had undergone an invasive procedure for CVD (coronary artery bypass graft, angioplasty, valve replacement, pacemaker placement, or other vascular surgeries), were excluded from the study at baseline (2000–2002). This study was approved by the Institutional Review Boards of each study site, and written informed consent was obtained from all participants.

The Health, Aging and Body composition (Health ABC) study is a longitudinal, prospective study investigating the associations among body composition, weight-related Health conditions, and incident functional limitations in older adults [25]. The Health ABC study cohort consists of 3,075 well-functioning black and white men and women aged 70–79 at baseline (1997-1998). After excluding participants with missing data and prevalent cardiovascular disease at baseline, 2,159 participants were analyzed for 8.6 years for incident CVD events.

### 2.2. Laboratory and Anthropometric Measurements

Participants completed standardized medical history questionnaires. Fasting blood glucose and lipids were analyzed at a central laboratory. Serum glucose was measured by Vitros analyzer (Johnson & Johnson Clinical Diagnostics). Impaired fasting glucose was defined as a fasting blood glucose ≥100 mg/dL and <126 mg/dL. Diabetes was defined by self-reported history of adult onset diabetes, fasting glucose ≥126 mg/dL, or use of insulin or oral glucose-lowering medications. Plasma lipids (HDL cholesterol and triglycerides) were measured using a standardized kit (Roche Diagnostics). LDL cholesterol was calculated with the Friedewald equation [26]. Resting seated blood pressure was measured three times using an automated oscillometric sphygmomanometer (Dinamap PRO 100; Critikon, Tampa, FL). The average of the last two measurements was used in analysis. Elevated blood pressure was defined by use of blood pressure medication or systolic blood pressure ≥130 mm Hg or diastolic blood pressure ≥85 mm Hg. Waist circumference was measured at the umbilicus to the nearest 0.1 cm using a steel measuring tape with standard 4-oz tension.

The National Cholesterol Education Program/Adult Treatment Panel (NCEP ATP III) [1, 9] definition was used to classify participants having MetS in the MESA cohort. Three of five components are required for diagnosis. (1) Waist circumference ≥ 102 cm: men, ≥88 cm: women, (2) hypertension ≥ 130 mm Hg systolic or ≥85 mm Hg diastolic or use of medications for hypertension, (3) fasting blood glucose ≥ 100 mg/dL or treatment for impaired fasting glucose, (4) triglycerides ≥ 150 mg/dL or specific treatment, (5) HDL-C ≤ 40 mg/dL in men and ≤50 mg/dL in women.

### 2.3. Cardiovascular Events

A detailed description of events and the process of adjudication can be found at the MESA website (http://www.mesa-nhlbi.org/). Briefly, participants were contacted every 9–12 months to inquire about hospital admissions, cardiovascular diagnoses, and deaths. Hospital records were abstracted for possible CVD events and were sent for review and classification by an independent adjudication committee. For the purposes of this study, a CVD event was defined as incident myocardial infarction, resuscitated cardiac arrest, definite angina, probable angina if followed by revascularization, stroke, stroke death, coronary heart disease (CHD) death, other atherosclerotic death, and other CVD deaths as defined by the MESA protocol.

### 2.4. Statistical Analysis

An ANOVA test was performed to compare the mean values of the components of the MetS across the four ethnic groups. A chi-square test was performed to compare the proportion of individuals exceeding the NCEP cut points for each component of the MetS across the four ethnic groups. A principal component analysis using studentized residuals of fasting log glucose, log triglycerides, log HDL cholesterol, waist circumference, systolic blood pressure, and diastolic blood pressure after adjusting for age and gender was performed. A correlation matrix was generated to measure the correlation between the individual elements of the metabolic syndrome and the first principal component. Principal component analysis was used for the extraction of the initial factors. Principal component analysis transforms the original variables into a new set of uncorrelated factors (principal components) that account for the maximum proportion of the variance in the data, with each component being a linear combination of the original observed variables. The first principal component is the linear combination of variables that accounts for the largest proportion of variance in the data, and the second component is the combination that accounts for the next largest proportion, and so on. Only components with eigenvalues (the sum of the squared factor loadings, representing the variance attributable to each principal component) >1.0 are considered significant. A Kaplan-Meier survival analysis was performed across quartiles of MetS-PC to predict CVD events and a log rank test for trend overall and in each ethnic group was performed. After excluding subjects with missing data, a Cox regression proportional hazards analysis was used to determine the association between MetS-PC and 5.5-year incident CVD events in the MESA cohort after adjusting for potential confounders including age, gender, race, smoking, and LDL-C in nested models.

In a separate sensitivity analysis, participants with diabetes were excluded, the first principal component was recalculated, and the association between MetS-PC and CVD events was performed. Similar sensitivity analyses were performed after excluding participants on anti-hypertensive medications and anti-lipid medications. The patterns of association observed between MetS-PC and CVD events in these sensitivity analyses were qualitatively similar to the results observed when using the first principal component generated using the entire cohort (sensitivity analyses data not shown), and thus the results for the entire cohort are shown.

In a separate analysis, MetS-PC was recalculated using the original six variables plus high-sensitivity C-reactive protein (hsCRP). This new MetS-PC with seven components was included in separate Cox proportional hazards models with potential confounders to predict incident CVD events.

To facilitate comparison of MetS-PC with the dichotomous NCEP definition, a MetS-PC cut-point (0.475) was chosen to yield the same 37% prevalence of metabolic syndrome as when using the NCEP definition (37%) in the MESA cohort. Separate Cox regression proportional hazards models including NCEP MetS and a binary MetS-PC to predict cardiovascular events were developed to compare the relative strength of the association of the two MetS definitions, and their model chi-square values were compared.

Similar analyses were performed using the Health ABC cohort to replicate and validate the findings from the MESA cohort. All statistical analyses were performed using JMP Version 8 (SAS Institute Inc., Cary, North Carolina).

## 3. Results

### 3.1. Participant Characteristics

Among 6,814 MESA participants, we excluded 34 due to missing MetS component data. The baseline characteristics of the 6,780 participants are shown in Table 1. There are significant differences in the prevalence of the elements of the MetS between the ethnic groups. The mean age of the participants in each ethnicity is 62 years, and more than 50% are females in each ethnicity. The first principal component explains 33% of the total variance among these measures, and the measured correlations are: waist circumference (0.56), systolic blood pressure (0.61), diastolic blood pressure (0.56), glucose (0.44), high-density lipoprotein (−0.51), and triglycerides (0.51). (Table 2) Consistent with the notion of a syndrome with highly correlated measures, all MetS-PC correlation coefficients were above 0.40, and the eigenvalue for the first principal component is 2.0.

### 3.2. Cardiovascular Events

**Figure 1:**
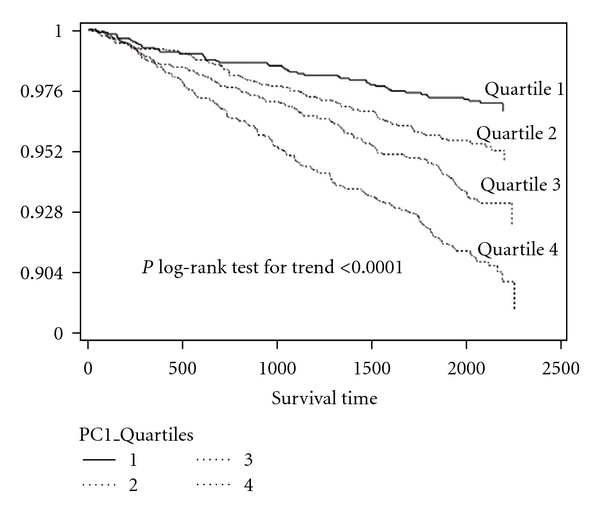
Kaplan-Meier survival curves for CVD events across quartiles of MetS-PC in MESA cohort after 5.5 years of followup. MetS-PC: quartile 1 < −0.96, quartile 2 > −0.96 to <−0.02, quartile 3 > −0.02 to <0.93, and quartile 4 > 0.93.

During 5.5 years of followup, 377 CVD events were identified (160 Caucasian, 30 Chinese, 105 African, and 82 Hispanic). A Kaplan-Meier survival curve shows decreased survival free of CVD events across quartiles of MetS-PC with a log test for trend which is statistically significant (*P* < 0.0001) (Figure 1). After stratification by ethnicity, similar results were obtained for each ethnicity separately (Chinese: *P* = 0.02, all other ethnicities: *P* < 0.001).

When treated as a continuous measure, each unit difference of standard deviation (SD) of MetS-PC was significantly associated with CVD events with a univariate hazard ratio of 1.56, 95%CI 1.42–1.72 (Table 3). In a full model adjusted for age, gender, race, smoking, and LDL-C, MetS-PC per unit difference of SD remained significantly associated with CVD events (HR = 1.71, 95%CI 1.54–1.90). Additionally in similar analysis stratified by ethnicity, hazard ratio remained significant in each ethnicity separately in both unadjusted and adjusted models (Table 3).

The prediction of CVD events using MetS-PC with seven components including hsCRP was not significantly different than the more parsimonious MetS-PC definition with the original six variables (data not shown).

Finally, when using a binary definition of MetS-PC (cut point 0.475) designed to match the NCEP definition in terms of prevalence in the MESA cohort, the fully adjusted HR for CVD events was 2.34, 95%CI 1.91–2.87 compared with 1.79, 95%CI 1.46–2.20 when using the NCEP definition (Table 4). A similar pattern of improved prediction of CVD events using the binary MetS-PC definition compared with the NCEP definition was also observed in each ethnicity separately. The sensitivity and specificity of the binary MetS-PC definition for prediction of CVD was 54% and 65%, respectively, compared with 51.7% and 63.5% using the NCEP definition. The formula for calculating MetS-PC is ((−11.8769) + (1.5432298* Log Glucose) + (0.7872732 * Log Triglyceride) − (1.588791 * Log HDL) + (0.0277125 * waist circumference) + (0.0232299 * SBP) + (0.0420722 * DBP) − (0.016408 * Age) − (0.73821 * Gender) Male = 1, Female = 0).

Finally, we performed an independent validation of the analysis using the Health ABC cohort. In a full model adjusted for age, gender, race, smoking, and LDL-C, MetS-PC per 1-SD (HABC) remained significantly associated with CVD events (HR = 1.21, 95%CI 1.12–1.32) overall, and by each ethnicity, Caucasian (HR = 1.24, 95%CI 1.12–1.39) and African Americans (HR = 1.16, 95%CI 1.01–1.32). Finally, when using a binary definition of MetS-PC (cut point 0.505) designed to match the NCEP definition in terms of prevalence in the Health ABC cohort, the fully adjusted HR for CVD events was 1.39, 95%CI 1.17–1.64 compared with 1.46, 95%CI 1.23–1.72 when using the NCEP definition.

## 4. Discussion

In this ethnically diverse population of 6,780 individuals aged 45–84, the first principal component derived from elements of the MetS explains 33% of the variance of the metabolic measures. This continuous metabolic syndrome score (MetS-PC) is a significant predictor of 5.5-year incident clinical cardiovascular events in the total population, and in each of the four major race/ethnicity subgroups separately. Additionally, a binary definition of the MetS based on this continuous measure was a better predictor of clinical cardiovascular events compared to the NCEP definition.

It should be acknowledged that MetS is not an absolute risk indicator, because it does not contain many of the factors that determine absolute risk, for example, age, sex, cigarette smoking, and low-density lipoprotein cholesterol levels [6]. Risk predictors such as the Framingham risk score are much superior if risk prediction is the goal; however, the primary goal of this analysis was not risk prediction per se, but rather improving upon the current NCEP definition of MetS, using the same components that make the MetS and comparing the two definitions using incident CVD events as a criteria measure.

The current binary definitions of the metabolic syndrome, including the NCEP definition, were developed by expert committees as a clinically useful means of identifying high-risk individuals [1, 3–6]. However, dichotomization of the continuous variables leads to loss of information [7, 8]. A minor improvement in one component could result in an individual no longer being classified as having MetS, despite no meaningful change of actual cardiovascular risk [11]. Additionally, the NCEP MetS may not predict risk of CVD events in all ethnicities equally as the data for constructing the NCEP definition were derived from predominantly Caucasian populations [12–16]. The evaluation of the MetS as a continuous score derived from a multiethnic population is potentially a more informative and generalizable approach to defining the syndrome and determining its clinical correlates. Additionally, a continuous measure may also improve the ability to identify lifestyle, environmental, molecular, and genetic etiologic factors that are specific for the MetS, and in future, these etiological factors could be incorporated in the definition of the continuous measure providing a stronger CVD predictive value [7].

Principal component analysis is a mathematical technique that transforms a number of correlated variables into a reduced number of uncorrelated variables called principal components, of which the first principal component captures the maximal variance [17, 18, 27]. Since the MetS is a metabolic condition characterized by the co-occurrence of multiple metabolic abnormalities, it follows that the first principal component of the measures for these traits would be an efficient way to quantify the presence of the syndrome. In our data, MetS-PC correlated well with all the components of the MetS (correlation coefficients 0.44–0.61, Table 2), consistent with a definition of a syndrome.

The metabolic syndrome is a known risk factor for cardiovascular disease, and MetS-PC predicts clinical cardiovascular events extremely well in this multiethnic cohort. MetS-PC was significantly associated with a more than twofold, fully adjusted increased risk of incident CVD in this cohort. We choose to show the analysis in the entire cohort including also diabetics as the analysis after excluding diabetics was qualitatively similar to the results obtained with the entire cohort. In analyses stratified by ethnicity, MetS-PC is a significant predictor of CVD across the four ethnic groups with similar point estimates for the hazard ratios. Additionally, there are suggestions that perhaps for better risk prediction; the definition of metabolic syndrome should be broadened to incorporate other elements such as high-sensitivity C-reactive protein (hsCRP) in the MetS definition [7, 28]. We performed an analysis wherein we added hsCRP in the MetS-PC, but the addition did not lead to any substantial improvement in risk prediction of incident CVD events. Our findings parallel similar results where addition of hsCRP to MetS did not lead to improvement in prediction of incident CVD events [29] or atherogenesis [30].

Further, we compared the MetS-PC definition of MetS with the NCEP definition in predicting CVD events in the MESA cohort. To facilitate comparison of MetS-PC with the dichotomous NCEP definition, a MetS-PC cut point (0.475) was chosen to yield the same 37% prevalence of MetS as when using the NCEP definition (37%) in the MESA cohort. The strength of association with incident cardiovascular events was compared using the NCEP and the derived binary MetS-PC definitions, in a fully adjusted model. The binary MetS-PC has a stronger association with incident cardiovascular events compared to the NCEP MetS as the point estimate for hazard using the binary MetS-PC definition is not included in the confidence interval of the NCEP hazard ratio. Additionally, the chi-square values were much higher when using the binary MetS-PC definition compared to the NCEP MetS definition. However, when an independent validation of a similar strategy was applied in the Health ABC cohort, the association of MetS-PC with CVD events remained, but the superiority of the MetS-PC definition versus NCEP MetS definition was no longer evident. This could be the result of the inherent differences between the two cohorts such as differences in age. The mean age of the MESA cohort is 62 years versus 74 for the Health ABC cohort. Additionally, the overall mean age and gender-adjusted systolic and diastolic blood pressures are much lower in the MESA cohort as compared to the Health ABC cohort. This is reflected in the loading for the first principal component. Both the systolic and diastolic blood pressures are well represented in the first principal component in the MESA cohort (loading factors: systolic BP = 0.61, diastolic BP = 0.56). In contrast, the systolic and diastolic BP were less heavily represented in the PC analysis for the Health ABC cohort (loading factors: systolic BP = 0.13, diastolic BP = 0.05), perhaps secondary to the greater prevalence of essential hypertension uncorrelated with the other metabolic derangements of the Met syndrome. In a previous Health ABC study evaluating CVD risk in older adults with MetS without past history of coronary heart disease and heart failure, the proportion of MI (6.1% versus 4.8%, *P* = 0.18) and HF hospital stay (5.6% versus 4.3%, *P* = 0.17), although higher among those with MetS compared to those without MetS, did not reach statistical significance [31]. This finding perhaps explains the lower hazards for CVD events in the Health ABC cohort compared to the MESA cohort findings.

 A few studies have applied PCA to identify factors and their relationship to incident diabetes and cardiovascular disease (CVD) using the elements of the MetS, measures of obesity, and insulin resistance [19–22]. Hillier et al., [22] found increased odds of cardiovascular events in 5,024 middle-aged French cohort; men, 1.7 (1.4, 2.1), and women 1.7 (1.0, 2.7), which is similar to our findings of 1.71 (1.54, 1.90). Lempiainen et al., [19] applied factor analysis to 1069 subjects 65 to 74 years old from Finland followed for a period of 7 years and found similar hazards of coronary events. Similarly, Pyörälä et al., [23] applied factor analysis to 970 healthy men aged 34 to 64 years in the Helsinki Policemen Study and found that the insulin resistance factor increased the hazard for coronary heart disease to 1.28 (95% CI 1.10–1.50) during 22 years of followup. The current study builds on these earlier efforts by applying similar methods to a larger and much more ethnically diverse cohort. The strength of the association between the continuous MetS-PC score and CVD events, its utility for predicting CVD events in multiple ethnicities, and its improved performance relative to the NCEP definition provide compelling evidence that this approach may be a superior strategy for defining the metabolic syndrome.

The strength of this study is the inclusion of four different ethnicities from six different recruitment sites in the United States, and stringent quality control procedures, as well as an average 5.5-year followup for identification of incident cardiovascular events. Additionally, we performed a replication of the strategy using an independent cohort and found results to be qualitatively similar. Among its limitations, the exclusion of individuals with known cardiovascular disease calls for caution in generalizing results to the total population. It should also be emphasized that acculturation to diet and environment plays a role in the development of the cardiovascular disease, and therefore, our findings may not apply to the same ethnicities in other parts of the world. Additionally, the sample size for Chinese Americans was small, and the event rate was low, making the estimates of risk less certain in the Chinese American participants compared with other groups. Further, this continuous score will need to be validated in larger cohorts before finding general applicability.

## 5. Conclusion

In this multi-ethnic population, the first principal component derived from the elements of the metabolic syndrome represents a continuous metabolic syndrome score which significantly predicts clinical cardiovascular events overall and across all ethnicities. Additionally, when a binary score derived from the first principal component was compared to the NCEP definition of MetS in the MESA cohort, the binary MetS-PC score was a better predictor of incident cardiovascular events than the NCEP definition of the metabolic syndrome. However, in the Health ABC cohort, although results were qualitatively similar, the MetS-PC definition was not found to be superior to the NCEP definition of MetS. This strategy will need to be replicated in other large multi-ethnic cohorts and, if also predictive of incident diabetes, it could be considered as an alternative to the conventional metabolic syndrome definition. More research will be required to clarify if and how such a score could be incorporated into clinical practice.

## Figures and Tables

**Table 1:** Baseline Characteristics of the MESA cohort (2000–2002).

Race/ethnicity	Caucasian	Chinese	African	Hispanic	*P* value
Participants	2610	801	1875	1494	
Age	62.6 (10.2)	62.3 (10.3)	62.1 (10.1)	61.3 (10.3)	0.0009
Male	1259 (48%)	390 (48%)	843 (45%)	721 (48%)	0.06
Glucose ≥ 100 mg/dL	835 (32%)	380 (47%)	853 (45%)	723 (48%)	0.0001
SBP ≥ 130 mm Hg	909 (35%)	303 (38%)	946 (50%)	596 (40%)	0.0001
DBP ≥ 85 mm Hg	195 (7%)	89 (11%)	285 (15%)	142 (9%)	0.0001
HDL ≤ 40, 50 mg/dL (M, F)	947 (36%)	347 (43%)	694 (37%)	740 (49%)	0.0001
Triglyceride ≥ 150 mg/dL	803 (31%)	276 (34%)	294 (16%)	638 (43%)	0.0001
Waist circ ≥ 102, 88 cm (M, F)	1409 (54%)	199 (25%)	1194 (63%)	921 (62%)	0.0001
Metabolic syndrome	862 (33%)	247 (31%)	721 (38%)	703 (47%)	0.0001
Fasting blood glucose (mg/dL)	98.4 (21.9)	106.1 (28.8)	107.2 (32.6)	110.8 (39.9)	0.0001
Systolic blood pressure (mm Hg)	123.5 (20.4)	124.6 (21.6)	131.7 (21.6)	126.7 (21.9)	0.0001
Diastolic blood pressure (mm Hg)	70.2 (9.9)	71.9 (10.3)	74.5 (10.2)	71.5 (10.1)	0.0001
High density lipoprotein (mg/dL)	52.2 (15.7)	49.5 (12.7)	52.4 (15.3)	47.6 (13.1)	0.0001
Triglyceride (mg/dL)	132.9 (90.2)	142.7 (84.8)	104.8 (68.6)	157.1 (101.1	0.0001
Waist circumference (cm)	97.9 (14.4)	87.1 (9.9)	101.2 (14.7)	100.6 (13.1)	0.0001
Hypertension	931 (36%)	311 (39%)	965 (51%)	606 (41%)	0.0001
Diabetes	140 (5%)	85 (11%)	300 (16%)	248 (17%)	0.0001
High cholesterol	1065 (41%)	286 (36%)	665 (35%)	522 (35%)	0.0003
Antihypertensive medications	691 (26%)	223 (28%)	911 (48%)	442 (30%)	0.0001
Diabetes medications	111 (4%)	77 (10%)	263 (14%)	214 (14%)	0.0001
Lipid medications	460 (18%)	118 (15%)	312 (16%)	211 (14%)	0.02
Never smokers	1157 (44%)	604 (75%)	850 (45%)	807 (54%)	
Former smokers	1157 (44%)	153 (19%)	691 (36%)	486 (32%)	0.0001
Current smokers	301 (11%)	45 (6%)	338 (18%)	203 (14%)	

*P* values obtained by one way analysis of variance. Data presented in total numbers (percentages) and continuous measures presented as mean value (standard deviation). SBP, systolic blood pressure, mm Hg; DBP, diastolic blood pressure, mm Hg; HDL mg/dL: high density lipoprotein cholesterol; NCEP: metabolic syndrome criteria (3 or more are required for diagnosis. (1) waist circumference ≥ 102 cm: men, ≥88 cm: women, (2) hypertension ≥ 130 mm Hg systolic or ≥85 mm Hg diastolic, (3) fasting blood glucose ≥ 100 mg/dL or treatment for impaired fasting glucose or diabetes, (4) triglycerides ≥ 150 mg/dL or specific treatment, and (5) HDL-C < 40 mg/dL in men and <50 mg/dL in women).

**Table 2:** The correlation between the original elements of metabolic syndrome and MetS-PC (first principal component).

MetS components	MetS-PC
Waist circumference	0.56 (0.54,0.58)
Systolic blood pressure	0.61 (0.60,0.63)
Diastolic blood pressure	0.56 (0.54,0.57)
Glucose	0.44 (0.42,0.45)
High-density lipoprotein	−0.51 (−0.53,−0.49)
Triglyceride	0.51 (0.49,0.53)

**Table 3:** Cox proportional Hazard models illustrating the association of MetS-PC per change in one SD with CVD events in the MESA cohort after 5.5 years of follow up overall and by race/ethnicity.

	HR	95% CI	*P* value
Model 1			
Overall	1.56	1.42, 1.72	0.0001
Caucasian	1.59	1.37, 1.84	0.0001
Chinese	1.42	0.99, 2.01	0.06
African	1.46	1.21, 1.75	0.0001
Hispanic	1.84	1.48, 2.30	0.0001

Model 2			
Overall	1.65	1.49, 1.82	0.0001
Caucasian	1.61	1.38, 1.87	0.0001
Chinese	1.49	1.03, 2.15	0.04
African	1.61	1.34, 1.92	0.0001
Hispanic	1.92	1.54, 2.40	0.0001

Model 3			
Overall	1.71	1.54, 1.90	0.0001
Caucasian	1.64	1.39, 1.94	0.0001
Chinese	1.59	1.08, 2.35	0.02
African	1.67	1.37, 2.02	0.0001
Hispanic	2.10	1.66, 2.65	0.0001

Standardized MetS-PC; mean (SD), −0.00056 (1); model 1, univariate analysis; model 2, adjusted for age and gender; model 3, model 2, plus cigarette smoking and low-density lipoprotein; the overall hazards were further adjusted for race/ethnicity in models 2 and 3.

**Table 4:** Cox proportional hazard models illustrating the association of NCEP and binary MetS-PC MetS definitions with CVD events in the MESA cohort.

All cardiovascular events (*n* = 377)								
	All CVD	NCEP MetS			MetS-PC MetS			

	HR	95% CI	*P* value	*χ* ^2^ value	HR	95% CI	*P* value	*χ* ^2^ value
Model 1								
Overall	1.87	1.53, 2.28	0.0001	37	2.14	1.75, 2.62	0.0001	55
Caucasian	1.99	1.47, 2.71	0.0001	19	1.97	1.45, 2.68	0.0001	19
Chinese	1.49	0.72, 3.10	0.28	1	1.61	0.78, 3.35	0.19	2
African	1.74	1.18, 2.54	0.005	8	2.21	1.50, 3.27	0.0001	16
Hispanic	1.99	1.28, 3.11	0.002	9	2.88	1.81, 4.60	0.0001	20

Model 2								
Overall	1.78	1.45, 2.18	0.0001	31	2.32	1.89, 2.84	0.0001	66
Caucasian	1.85	1.36, 2.52	0.0001	15	1.98	1.45, 2.69	0.0001	19
Chinese	1.42	0.68, 2.99	0.35	1	1.71	0.82, 3.57	0.15	2
African	1.73	1.18, 2.55	0.005	8	2.66	1.79, 3.94	0.0001	24
Hispanic	1.86	1.19, 2.91	0.007	7	3.10	1.94, 4.96	0.0001	22

Model 3								
Overall	1.79	1.46, 2.20	0.0001	31	2.34	1.91, 2.87	0.0001	67
Caucasian	1.84	1.35, 2.51	0.0001	15	1.95	1.43, 2.67	0.0001	18
Chinese	1.50	0.71, 3.17	0.28	1	1.80	0.86, 3.77	0.12	2
African	1.75	1.19, 2.57	0.004	8	2.68	1.81, 3.98	0.0001	24
Hispanic	1.92	1.22, 3.03	0.005	8	3.38	2.09, 5.44	0.0001	25

Model 1, univariate analysis; model 2, adjusted for age and gender; model 3, model 2, plus cigarette smoking and low-density lipoprotein; the overall hazards were further adjusted for race/ethnicity in models 2 and 3; CVD, cardiovascular disease; NCEP MetS (binary variables); MetS-PC (binary variables, with the cut point being 0.475); HR hazards ratio.

## References

[1] Expert Panel on Detection E (2001). Executive summary of the third report of the national cholesterol education program (NCEP) expert panel on detection, evaluation, and treatment of high blood cholesterol in adults (adult treatment panel III). *Journal of the American Medical Association*.

[2] Reaven GM (1988). Role of insulin resistance in human disease. *Diabetes*.

[3] Alberti KGMM, Zimmet PZ (1998). Definition, diagnosis and classification of diabetes mellitus and its complications. Part 1: diagnosis and classification of diabetes mellitus. Provisional report of a WHO consultation. *Diabetic Medicine*.

[4] Balkau B, Charles MA (1999). Comment on the provisional report from the WHO consultation. European group for the study of insulin resistance (EGIR). *Diabetic Medicine*.

[5] Alberti KG, Zimmet P, Shaw J (2005). The metabolic syndrome—a new worldwide definition. *The Lancet*.

[6] Alberti KG, Eckel RH, Grundy SM (2009). Harmonizing the metabolic syndrome: a joint interim statement of the international diabetes federation task force on epidemiology and prevention; National heart, lung, and blood institute; American heart association; World heart federation; International atherosclerosis society; And international association for the study of obesity. *Circulation*.

[7] Kahn R, Buse J, Ferrannini E, Stern M (2005). The metabolic syndrome: time for a critical appraisal—joint statement from the American diabetes association and the European association for the study of diabetes. *Diabetes Care*.

[8] Simmons RK, Alberti KG, Gale EA (2010). The metabolic syndrome: useful concept or clinical tool? Report of a WHO expert consultation. *Diabetologia*.

[9] Grundy SM, Cleeman JI, Daniels SR (2005). Diagnosis and management of the metabolic syndrome: an American heart association/national heart, lung, and blood institute scientific statement. *Circulation*.

[10] Al-Barwani SA, Bayoumi RA, Jaju D (2008). Differing definition-based prevalence of metabolic syndrome in the women of oman family study: a function of multiparity. *Metabolic Syndrome and Related Disorders*.

[11] Balkau B, Vernay M, Mhamdi L (2003). The incidence and persistence of the NCEP (National Cholesterol Education Program) metabolic syndrome. The French D.E.S.I.R. study. *Diabetes & Metabolism*.

[12] Shah T, Jonnalagadda SS, Kicklighter JR, Diwan S, Hopkins BL (2005). Prevalence of metabolic syndrome risk factors among young adult Asian Indians. *Journal of Immigrant Health*.

[13] Enkhmaa B, Shiwaku K, Anuurad E (2005). Prevalence of the metabolic syndrome using the third report of the national cholesterol educational program expert panel on detection, evaluation, and treatment of high blood cholesterol in adults (ATP III) and the modified ATP III definitions for Japanese and Mongolians. *Clinica Chimica Acta*.

[14] Shiwaku K, Nogi A, Kitajima K (2005). Prevalence of the metabolic syndrome using the modified ATP III definitions for workers in Japan, Korea and Mongolia. *Journal of Occupational Health*.

[15] Clark LT, El-Atat F (2007). Metabolic syndrome in African Americans: implications for preventing coronary heart disease. *Clinical Cardiology*.

[16] Centers for Disease C (2009). Differences in prevalence of obesity among black, white, and Hispanic adults—United States, 2006–2008. *Morbidity and Mortality Weekly Report (MMWR)*.

[17] Jolliffe I (2002). *Principal Components Analysis*.

[18] Jackson J (1991). *A User's Guide to Principal Components Analysis*.

[19] Lempiainen P, Mykkanen L, Pyorala K, Laakso M, Kuusisto J (1999). Insulin resistance syndrome predicts coronary heart disease events in elderly nondiabetic men. *Circulation*.

[20] Kekäläinen P, Sarlund H, Pyörälä K, Laakso M (1999). Hyperinsulinemia cluster predicts the development of type 2 diabetes independently of family history of diabetes. *Diabetes Care*.

[21] Hanson RL, Imperatore G, Bennett PH, Knowler WC (2002). Components of the "metabolic syndrome" and incidence of type 2 diabetes. *Diabetes*.

[22] Hillier TA, Rousseau A, Lange C (2006). Practical way to assess metabolic syndrome using a continuous score obtained from principal components analysis. *Diabetologia*.

[23] Pyörälä M, Miettinen H, Halonen P, Laakso M, Pyörälä K (2000). Insulin resistance syndrome predicts the risk of coronary heart disease and stroke in healthy middle-aged men: the 22-year follow-up results of the helsinki policemen study. *Arteriosclerosis, Thrombosis, and Vascular Biology*.

[24] Bild DE, Bluemke DA, Burke GL (2002). Multi-ethnic study of atherosclerosis: objectives and design. *American Journal of Epidemiology*.

[25] Harris TB, Visser M, Everhart J (2000). Waist circumference and sagittal diameter reflect total body fat better than visceral fat in older men and women. The health, aging and body composition study. *Annals of the New York Academy of Sciences*.

[26] Friedewald WT, Levy RI, Fredrickson DS (1972). Estimation of the concentration of low-density lipoprotein cholesterol in plasma, without use of the preparative ultracentrifuge. *Clinical Chemistry*.

[27] Cureton EE DAR (1983). *Factor Analysis: An Applied Approach*.

[28] Ridker PM, Wilson PW, Grundy SM (2004). Should C-reactive protein be added to metabolic syndrome and to assessment of global cardiovascular risk?. *Circulation*.

[29] Rutter MK, Meigs JB, Sullivan LM, D’Agostino RB, Wilson PW (2004). C-reactive protein, the metabolic syndrome, and prediction of cardiovascular events in the Framingham offspring study. *Circulation*.

[30] Reilly MP, Wolfe ML, Rhodes T, Girman C, Mehta N, Rader DJ (2004). Measures of insulin resistance add incremental value to the clinical diagnosis of metabolic syndrome in association with coronary atherosclerosis. *Circulation*.

[31] Butler J, Rodondi N, Zhu Y (2006). Metabolic syndrome and the risk of cardiovascular disease in older adults. *Journal of the American College of Cardiology*.

